# Which nostril should be used for nasotracheal intubation with Airtraq NT®: the right or left? A randomized clinical trial

**DOI:** 10.3906/sag-1803-177

**Published:** 2019-02-11

**Authors:** Zehra İpek ARSLAN, Neşe TÜRKYILMAZ

**Affiliations:** 1 Associate Professor, Kocaeli University Medical Faculty, Anesthesiology and Reanimation, Kocaeli Turkey; 2 Specialist, Kocaeli University Medical Faculty, Anesthesiology and Reanimation, Kocaeli Turkey

**Keywords:** Airtraq, nasotracheal, right nostril, 90° degree counterclockwise, cricoid pressure, head flexion

## Abstract

**Background/aim:**

Nasotracheal Airtraq is specifically designed to improve the glottis view and ease the nasotracheal intubation process in normal and difficult cases.

**Materials and methods:**

After Ethics committee approval, we decided to enroll 40 patients with an ASA physical status of I or II, between 18 and 70 years of age undergoing elective maxillofascial, oral, and double chin surgery to determine which nostril is more suitable for nasotracheal intubation with nasotracheal Airtraq. Patients were randomized into the right and left nostril groups.

**Results:**

Demographic and airway characteristics were similar among the groups. Nasotracheal intubation through the right nostril was shorter than that of the left nostril during nasotracheal intubation with the Airtraq NT (P < 0.001). 90° counterclockwise rotation of the tip of the tube was needed for directing the tube into the vocal cords in both right and left nostril groups (72% vs 88%). External laryngeal pressure and head flexion maneuvers can ease the intubation from the left nostril (P < 0.001 vs P = 0.03). Cuff inflation maneuver also can be helpful in some cases. We did not need any operator change or Magill forceps for any of the patients.

**Conclusion:**

Nasotracheal intubation via the right nostril can be safely and quickly performed with the Airtraq NT without the need of Magill forceps. We recommend the use of the 90° counterclockwise rotation, external laryngeal pressure, and head flexion maneuvers to direct the tube into the vocal cords first. On the other hand, cuff inflation maneuver must also be kept in mind.

## 1. Introduction

Anesthetists have been used to performing nasotracheal intubation during their daily practice since Kuhn’s first description of it in 1902 (1). Since the 1920s, nasotracheal intubation usually has been performed with the Macintosh and the aid of the Magill forceps (2). However, Magill forceps can lead to the rupture of the cuff or mucosal injury. Although fiberoptic intubation is the gold standard, it is hard to use it in every nasotracheal intubation and it requires technical skill and takes more time than with the Macintosh or videolaryngoscopes. 

**Figure 1:**
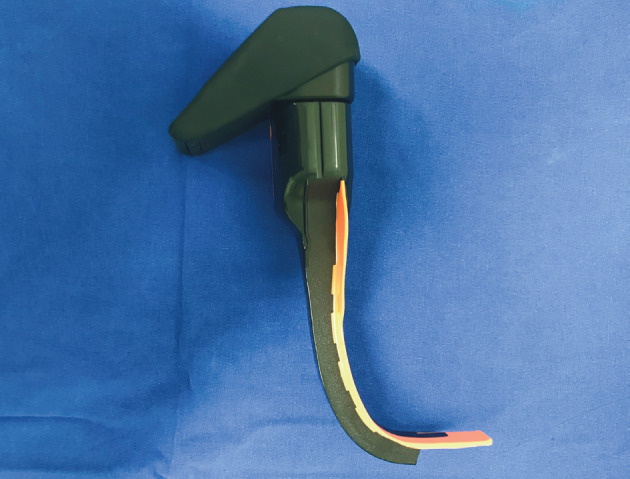
Nasotracheal Airtraq with and attachable high resolution Wi-Fi camera.

Nasotracheal Airtraq (Airtraq NT; Prodol Meditec S.A., Vizcaya, Spain) is an optical laryngoscope that was specifically designed for nasotracheal intubation procedure without a channel (Figure 1). A new design of video laryngoscopes improved the Cormack–Lehane grade of the patients and allows for nasotracheal intubation without the need of Magill forceps (3,4). 

When performing nasotracheal intubation anesthetists selected the more patent nostril or the nostril that the surgeon preferred. A recently published article forced us to think about the possible association between the successful intubation via the right nostril using a multiplanar computed tomography (5).

**Figure 2:**
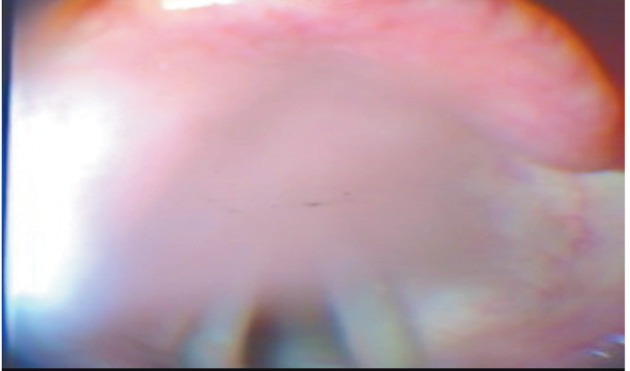
Baseline glottic view of Airtraq NT from the right nostril. The tube is extremely at the right bottom.

**Figure 3:**
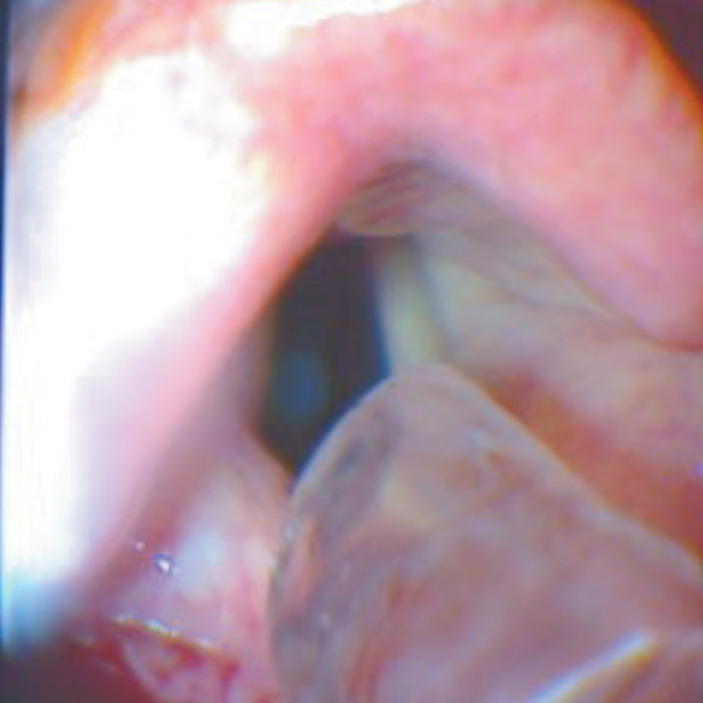
90° counterclockwise rotation of the tube helps us to view the tip of the tube and direct it into the center of glottis inlet.

**Figure 4:**
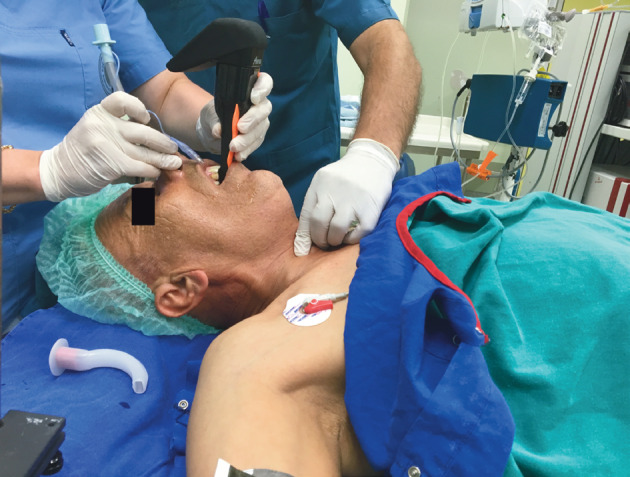
External laryngeal pressure maneuver to direct the tube into the vocal cords.

**Figure 5:**
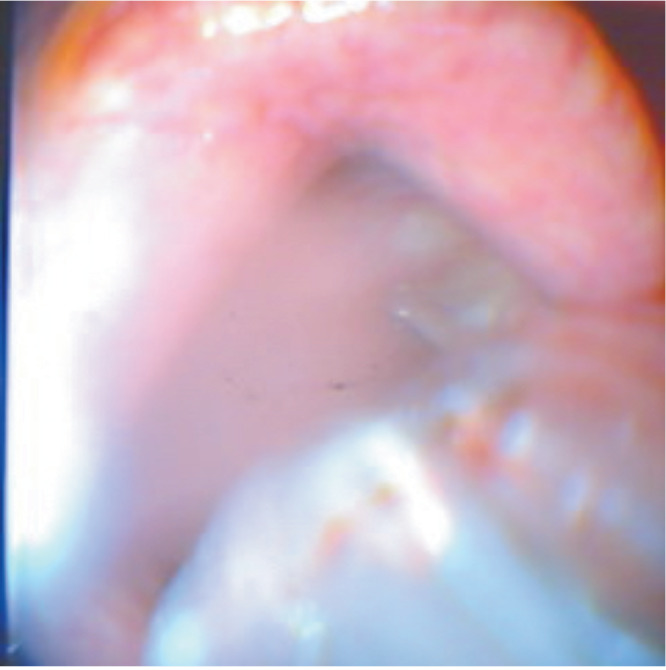
Cuff inflation maneuver to direct the tube into the vocal cords.

**Figure 6:**
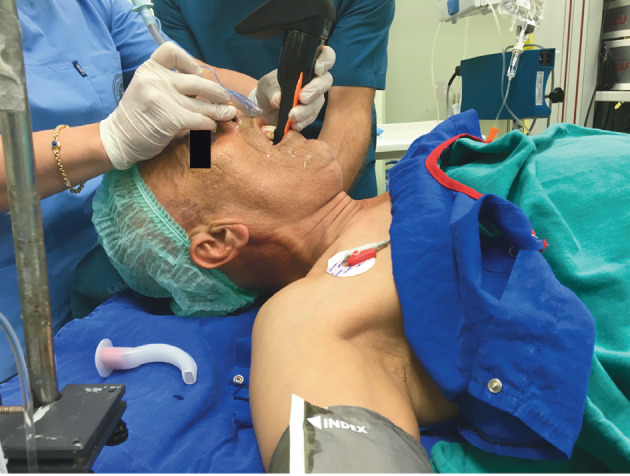
Head flexion maneuver to direct the tube into the vocal cords.

Starting from the previously published literature regarding the use of the 90° counterclockwise rotation of the tip of the tube as a technique being helpful for the insertion of the tube into the trachea during fiberoptic intubation and a later one that recommended orotracheal intubation with the Aitraq NT using this maneuver, we decided to use this maneuver first when we encountered any difficulty while inserting the tube into the trachea during nasotracheal intubation with the Airtraq NT (6-9)(Figure 2,3). Then the external laryngeal pressure (Figure 4), then cuff inflation (Figure 5), head extension (Figure 6), operator change, and Magill’s forceps were used in random order (10). 

The primary aim of this prospective randomized study was to compare the right and the left nostrils regarding the insertion and intubation times. Secondary aim was to report the need for the 90° counterclockwise rotation, external laryngeal pressure, tube cuff inflation and head flexion and changing the operator maneuvers without using Magill forceps to direct the tube through the vocal cords during nasotracheal intubation with the Airtraq NT.

## 2. Materials and methods 

This study was approved by the Local Human Research Ethics Committee. Additionally, this trial was also registered at ClinicalTrials (NCT03226002). After the written informed consent was obtained from each patient, 40 patients’ ASA physical status I-II, between 18 and 70 years of age, undergoing elective maxillofacial, oral, orthognatic surgery (double chin surgery) requiring nasotracheal intubation were enrolled in this prospective randomized study. Patients with a history of difficult intubation, limited mouth opening <3 cm, full stomach, and upper respiratory tract infection were excluded from this study. The patients were premedicated with midazolam 0.03 mg kg−1 intravenously (iv) at preoperative care unit which anesthesia nurse monitored and premedicated the surgical patients. 

When patients arrived in the operating room, the standard anesthesia monitoring including ECG, noninvasive blood pressure, heart rate, pulse oximetry, and end-tidal carbon dioxide were applied. The patients were randomized into two groups; the right and the left nostril groups using a sealed envelope technique. Demographic (age, sex, weight, height, ASA physical status) and airway variables (thyromental distance, sternomental distance, interincisor distance, mallampati, head extension, mandibular protrusions (A; lower incisors protruded more than upper incisors, B; lower incisors can be brought edge to edge with the upper incisors, C; lower incisors cannot be brought to the upper incisors), teeth morphology (full/lack/absent)) were recorded. Types of surgery were also recorded. 

General anesthesia was induced with propofol 3 mg kg−1 iv and fentanyl 1 mg kg−1 iv. Ease of facemask ventilations were recorded as easy, airway, two-handed, oxygen flush, and impossible (11). Then, 0.6 mg kg−1 of iv rocuronium was administered for muscle relaxation. First, we inserted the cuffed spiral lateral beveled endotracheal tube (Mallinckdrodt Medical, Athlone, Ireland) lubricated with lidocaine jelly into the selected nostril with a diameter of 7.5 mm for men and 7.0 mm for women. Then, if any resistance was felt, we tried the other nostril and then changed the tube with a small one to the selected nostril in random order. Following the insertion of the spiral tube through the nostril, we started to intubate the patient with the Airtraq NT. We recorded the insertion time, nasotracheal intubation time, total nasotracheal intubation time, and Cormack–Lehane grades of the patients. 

Insertion time is the time elapsing from the device entering the oral cavity until the optimal glottis visualization occurred. Reinserting and handling force maneuver included in this time period. Nasotracheal intubation time is the time elapsing from the device entering the oral cavity until the visualization of the tube entering through the vocal cords. When a resistance was felt during the tube adjustment, the maneuvers included 90° counterclockwise rotation maneuver (6,12), external laryngeal pressure (3,13), cuff inflation (14,15), head flexion, changing the operator, use of Magill forceps were applied (10) in random order. Total nasotracheal intubation time is the time elapsing from the device entering the oral cavity until the confirmation of intubation from the capnograph.

Systolic blood pressure, diastolic blood pressure, mean arterial pressure, heart rate, and pulse oximetry were recorded baseline (preoperatively), after anesthesia induction, and 1 min intervals after intubation for 3 min by an independent unbiased observer. Occurrence of epistaxis was also recorded. In the case where the patient could not be intubated after three attempts or after 120 s, then she/he was intubated with the Macintosh laryngoscope and Magill forceps. All intubations were performed by individuals with at least 4 years of anesthesia experience, experience with orotracheal intubation with the Airtraq and who had at least 20 successful intubations with the Airtraq NT. SpO2 < 92 was recorded as hypoxemia. 

We based our sample size on our preliminary study. We enrolled 4 patients for each group, 8 patients in total for this calculation. We found total intubation times as 29.5 (2.38) for the right nostril and 44.3 (3.95) for the left nostril. Based on this data, a = 0.05 and b = 0.2, we needed 15 patients for each group. We decided to enroll 20 patients per group considering possible exclusions. Continuous data were examined for normal distribution with the Kolmogorov–Smirnov test. For normally distributed data, we used the Student’s t-test, and Mann–Withney U test for not normally distributed data. Normally distributed data were given as mean ± standard deviation (SD) and nonnormally distributed data as median [25–75 percentiles]. Categorical data was calculated with Chi-square test. P < 0.05 was considered statistically significant.

## 3. Results

**Figure 7:**
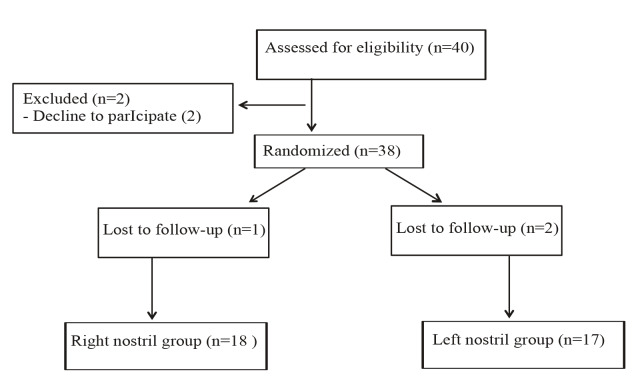
Flow chart.

We enrolled 40 patients; however, 2 patients denied participation in this study and 3 patients lost-to-follow-up. Therefore, we analyzed 35 patients (18 patients in the right nostril group, 17 patients in the left nostril group) (Figure 7).

Demographic and airway variables of the nostril groups were similar. Types of surgery and airway management parameters were similar between the groups (Table 1). Mallampati, mandibular protrusions, interincisor distance of patients with mandibular fractures could not be evaluated, so the values were not presented in Table 1. 

**Table 1:** Demographic and airway characteristics of patients; values were given as numbers (n), mean (SD), or IQR [25–75 percentiles].

	Right nostril (n = 18)	Left nostril (n = 17)	P
ASA I /II (n)	16 /2	14 /3	0.658
Sex F/M (n) Height (cm) Weight (kg) Age (years) Sternomental distance (cm) Tyromental distance (cm) Interincisor distance (cm) Mandibular protrusion A/B (n) Teeth morphology Full/lack/absent (n)	8 /10 172.3 (9.9) 72 (16.7) 25.5 [20.8-37] 14 [14-16.3] 8 [7-9] 4 [4-5] 13 /1 15 / 2 / 1	6 / 11 170.1 (8.7) 70.4 (14.7) 30 [23-52] 15 [14-16] 9 [7-9] 4.5 [4-5] 11 /0 14 / 1 / 2	0.836 0.474 0.759 0.869 0.909 0.130 0.610 0.366 0.714
Mallampati I / II / III / IV (n)	5 / 5 / 3 / 1	5 / 5 / 1 / 0	0.800

All the patients were intubated at the first attempt successfully in the right nostril group and all except one patient in the left nostril group. The reinserting maneuver was used with more than half of the patients in both groups for optimization of the glottis view of the Airtraq NT. There was no need to apply the handling force maneuver for optimization of the glottic view. Cormack–Lehane grades were similar between the groups (Table 1). 

The insertion times were comparable between the groups. Nasotracheal intubation and total nasotracheal intubation times were shorter in the right nostril group compared to the left nostril group (P < 0.001)(Table 2). No hypoxemia occurred during the entirety of the procedure. Especially the 90° counterclockwise rotation of the tip of the tube was needed to direct the tube into the vocal cords for both nasotracheal intubations of the right and left nostril groups (72% vs 88%)(Table 2). When this maneuver was felt, secondly, we applied external laryngeal manipulation (Sellick maneuver) to direct the tube into the vocal cords during nasotracheal intubation through the left nostril (P < 0.001) (Table 2). Head flexion maneuver was statistically significantly helpful while intubating through the left nostril (P = 0.03) (Table 2). Inflating the tube cuff was helpful in some cases (Table 2). We did not need any operator change, any use of Magill forceps. Hemodynamic variables were similar among the groups. 

**Table 2:** Nasotracheal intubation variables of patients, values were given as numbers (n), mean (SD), or IQR [25–75 percentiles].

	Right nostril (n = 18)	Left nostril (n = 17)	P
Mask ventilation Easy/airway/two-handed Number of intubation attempts (n) Reinserting maneuver Yes / No Insertion time (s)	11 / 5 / 2 18 / 0 6 / 12 5.8 (1.9)	11 / 2 / 4 16 / 1 8 / 9 6.5 (1.8)	0.447 0.486 0.629 0.293
Nasotraheal intubation time (s)	16.7 (5.7)	33.1 (15.4)	<0.001&
Total nasotracheal intubation time (s)	30 [28-31.3]	50 [33.5-62]	<0.001&
SpO2 (%) 90° counterclockwise rotation Yes / No (n) OELM Yes /No Head flexion Yes / No Tube cuff inflation Yes / No Epistaxis Yes / No	99.5 [98.8-100] 13 / 5 72% / 28% 0 / 18 0 / 18 1 / 17 3 / 15	99 [99-100] 15 / 2 88% / 12% 9 / 8 4 / 13 5 / 12 4 / 13	0.732 0.237 <0.001& 0.03* 0.06 0.612

* P < 0.05 and & P < 0.001

## 4. Discussion 

The main result of this study was that nasotracheal intubation could be performed in a shorter time through the right nostril than through the left nostril. In addition, 90° counterclockwise rotation maneuver of the endotracheal tube is useful for nasotracheal intubation for both right and left nostrils with the Airtraq NT. External laryngeal pressure and head flexion maneuvers eased the nasotracheal intubation from the left nostril. Cuff inflation could also be performed for nasotracheal intubation with the Airtraq NT instead of using Magill forceps. 

There are possible reasons for shorter intubation time when nasotracheal intubation was performed through the right nostril. First, the tube is often displaced to the right pharyngeal wall and it makes it easier to view the tip of the tube and manipulate it with the tube adjustment maneuvers or with the Magill forceps (16,17). Second, the multiplanar imaging studies showed that the ease was due to the association between the right nostril and the posterior nasopharyngeal anatomy (5). 

Consistent with our results, there was a recently published awake nasotracheal intubation case with an oral cancer with Mallampati IV using the Airtraq NT through the right nostril at the first attempt without the need of any maneuvers or Magill forceps (18). 

Gomez et al. (19) reported that anesthesia residents needed the external laryngeal pressure maneuver in 32% of difficult nasotracheal intubation of a manikin with the Airtraq NT. In addition, they had to use Magill forceps, stylet, or operator change in some difficult cases. Inexperienced users needed help with the maneuvers more often, the use of Magill forceps or operator change during the nasotracheal intubation process. Even in inexperienced hands, Airtraq NT had the fastest intubation, better Cormack–Lehane grades, and required less force than McGarth MAC and Macintosh in this study. The necessity of maneuvers (head flexion, external laryngeal pressure) to advance the endotracheal tube through the vocal cords was also taken into consideration. In our study, all nasotracheal intubations were performed with experienced users of both orotracheal AirtraqÒ and the Airtraq NT; we did not need any use of Magill forceps or operator change in this study. 

Some authors recommended the cuff inflation method to direct the tip of the tube to the vocal cords if the tube was felt excessively posterior or lateral to the glottis opening without using Magill forceps or bougie (13,15,20,21). We used the cuff inflation maneuver and it helped us to direct the tip of the tube through the vocal cords easily in some cases.

When nonanesthesiologist physicians (novice laryngoscopy professionals) performed nasotracheal intubation, they could intubate easily and faster with the Airtraq NT (65 s) than with the Macintosh and Magill forceps combination (22,23). 

Mont et al. (24) compared the Airtraq NT and the Macintosh laryngoscopes in both easy and difficult nasotracheal intubations in 200 patients. In the expected difficult nasotracheal intubation group; the Cormack–Lehane grades were better, intubation times were shorter and the number of optimization maneuvers were lower in the Airtraq NT group. There was no data about the nostril side in this manuscript. 30% of the expected easy nasotracheal intubation patients in the Airtraq NT group needed manipulations such as external laryngeal pressure, guidance of the Eschmann stylet, changes in the head position, and the use of Magill’s forceps. However, they did not perform these four maneuvers in a strict order. In the expected difficulty groups, the need for these maneuvers increased significantly. They allowed the anesthetists to change the order. However, we did them in order in our study. 

In a published prospective randomized study that compared the Airtraq NT and the Macintosh laryngoscope in routine nasotracehal intubation of 62 patients, it was reported that laryngoscopy, tube insertion, and intubation times were similar in both groups. However, intubation difficulty scales were significantly reduced in the Airtraq NT group compared to the Macintosh laryngoscope group. In the Airtraq NT group, external manipulation was done to direct the tube through the vocal cords. There was no data about the nostril selection in this trial either (25). 

There are few studies and even case reports published in difficult nasotracheal intubation situations. When we read them, we saw that all successful intubations were performed from the right nostril with the Airtraq NT and McGrath MAC and with help of the 90° counterclockwise rotation maneuver (7,18,26). 

The limitation of our study is as follows; the operators were not blind to the selected nostrils and our patients were not expected to be difficult intubation patients. Further investigations are needed in nasotracheal intubation patients with different videolaryngoscopes, different maneuvers that would be helpful advancing the tube into the vocal cords, different shaped tips of endotracheal tubes, in different difficult intubation conditions. Maybe, a left channeled Airtraq NT will be produced in the future to ease through the left nasotracheal intubation processes (6,9,27). 

In conclusion, we recommend the use of the right nostril with the help of the 90° counterclockwise rotation, external laryngeal pressure, and head flexion maneuvers to shorten the nasotracheal intubation process without the need of Magill forceps while using the Airtraq NT. 

## Acknowledgments

We thank Prodol for providing Nasotracheal Airtraq for this trial.
